# Links between Endothelial Glycocalyx Changes and Microcirculatory Parameters in Septic Patients

**DOI:** 10.3390/life11080790

**Published:** 2021-08-05

**Authors:** Egle Belousoviene, Inga Kiudulaite, Vidas Pilvinis, Andrius Pranskunas

**Affiliations:** Department of Intensive Care, Lithuanian University of Health Sciences, Eiveniu Street 2, 50161 Kaunas, Lithuania; egle.belousoviene@lsmuni.lt (E.B.); inga.kiudulaite@lsmuni.lt (I.K.); vidas.pilvinis@lsmuni.lt (V.P.)

**Keywords:** sepsis, glycocalyx, microcirculation

## Abstract

The glycocalyx is an endothelial surface layer that is essential for maintaining microvascular homeostasis. Impaired integrity of the endothelial glycocalyx may be directly related to the development of microvascular dysfunction. To explore this hypothesis, we conducted a prospective observational study on adult patients diagnosed with sepsis. The study aimed to evaluate the degree of damage to the glycocalyx and to identify correlations between microcirculatory parameters and glycocalyx thickness based on capillary diameter. Sublingual microcirculation was examined using a handheld Cytocam-incident dark field video microscope. A sidestream dark field video microscope attached to a GlycoCheck monitor was used to determine the perfused boundary regions (PBRs) of sublingual blood vessels grouped by diameter (5–9 μm, 10–19 μm, and 20–25 μm). We identified significant damage to the glycocalyx in sublingual blood vessels of all the aforementioned diameters in septic patients compared to healthy age-matched controls. Furthermore, we found that the PBRs of the smallest capillaries (diameter class 5–9µm) correlated moderately and inversely with both total and perfused blood vessel densities. Collectively, our data suggest that there may be a functional relationship between damage to the endothelial glycocalyx of the smallest capillaries and alterations in the microcirculation observed in response to sepsis.

## 1. Introduction

The endothelial glycocalyx (EG) is a component of the luminal layer of intact blood vessels that is composed of proteoglycans and glycosaminoglycans. Together with plasma proteins (primarily albumin), the EG forms an endothelial surface layer that is extremely important for maintaining microvascular homeostasis [1]. Impaired EG integrity may lead directly to changes in vascular function, including increased permeability, altered modulation of biochemical signals, and impaired hemostasis [2]. These pathophysiological responses all contribute to the pathogenesis of sepsis and associated organ failure. Shedding of the EG results in the exposure of adhesion molecules. Activation of these molecules leads to the recruitment of leukocytes and platelets and the development of intravascular thrombi, fibrin formation, vascular hyporeactivity, increased vascular permeability, and ultimately results in circulatory dysfunction [3,4]. 

The current published literature that addresses the relationship between EG degradation and various microcirculatory parameters associated with human sepsis is limited and controversial. Moreover, the importance of changes in the EG in vessels of varying diameters has not been fully addressed. The thickness (and potentially the constituents) of the EG possibly vary depending on the vessel size [4]. 

Most capillaries are less than 4–8 µm in diameter; many critical pathophysiologic processes may take place in these small vessels [5,6]. Furthermore, results from previous studies revealed a statistically significant decrease in vascular density in septic patients that was limited to blood vessels of 5, 6, and 7 μm (i.e., reductions of 63%, 42%, and 28%, respectively, compared to controls). By contrast, no changes were observed in the density of the larger blood vessels (8–25 μm) when comparing results from septic patients to controls [7].

Current evidence suggests a direct correlation between the degree of EG damage, the severity of illness, and mortality [8]. Increased mortality and development of organ dysfunction in septic patients have also been directly related to microcirculatory impairment [9,10]. Thus, we hypothesized that there is a direct relationship between the changes observed in the microcirculation and EG damage in blood vessels of varying diameters in response to sepsis. Our secondary aim was to verify the differences in EG thickness in blood vessels of various sizes in septic patients compared to healthy controls.

## 2. Materials and Methods

The results were obtained from a prospective observational study that was carried out at the Lithuanian University of Health Sciences Hospital Kaunas Clinics from 2018 through to 2021. The study was performed according to the Declaration of Helsinki and approved by the Kaunas Regional Biomedical Research Ethics Committee (BE-2-5). All participants or their legal representatives signed the informed consent form.

Adult patients with sepsis and septic shock were evaluated for enrollment in the study within 24 h after admission to the intensive care unit. Following the third international consensus, sepsis was diagnosed in patients exhibiting an acute increase of ≥2 in the Sequential Organ Failure Assessment (SOFA) score in whom infection was either documented or suspected. Septic shock was defined as sepsis in which vasopressor therapy was required to maintain mean arterial pressure (MAP) >65 mmHg together with a serum lactate concentration >2 mmol/L despite adequate fluid resuscitation [11].

Patients were excluded from the study under the following conditions: (1) informed consent could not be obtained, (2) the patient was pregnant or breastfeeding, or (3) the patient was moribund and not expected to survive for 24 h. Patients with damaged sublingual mucosa were also excluded from the study. The control group included 30 apparently healthy age-matched volunteers.

Demographic and physiological variables, including SOFA scores, Acute Physiologic Assessment and Chronic Health Evaluation (APACHE) II score, and routine laboratory parameters were obtained for each septic patient when sublingual videomicroscopy was performed.

### 2.1. Evaluation of the Microcirculation

Sublingual video microscopy is currently considered to be the gold standard for the evaluation of microcirculation [12]. Video images of the sublingual microcirculation were obtained using a handheld Cytocam incident dark field (IDF) video microscope (Braedius Medical, Huizen, The Netherlands). This tool was developed as a means to examine the microcirculation at organ surfaces. Vessel walls cannot be visualized using this device; vessels can be detected and evaluated only when they are filled with red blood cells (RBCs). The IDF imaging principle is based on the absorption of green light (wavelength 530 nm) emitted by the microscope by hemoglobin in the RBCs. RBCs are reflected as dark dots, thus permitting the user to visualize the capillaries [13]. Results from a recently published study concluded that microcirculation images from Cytocam IDF imaging are of higher quality than those resulting from sidestream dark field (SDF) imaging [14].

After gently removing saliva with an isotonic saline-drenched dressing, the microscope was applied to the sublingual mucosa. Image sequences from at least three sections were captured while avoiding artifacts due to pressure at the site. Trained certified investigators who were blinded to the source of the data used validated AVA 3.2 software (AVA, MicroVision Medical BV, Amsterdam, The Netherlands) to analyze video clips. The clips were arranged at random to avoid coupling. Expert recommendations [15] were followed for the quality and analysis of the recorded images.

The images were divided into four equal quadrants. The blood flow through each quadrant was quantified by visual inspection. Scores included 0, no flow; 1, intermittent flow; 2, sluggish flow; and 3, continuous flow for each vessel within a specific diameter cohort (i.e., types including small, 10–20 µm; medium, 21–50 µm; large, 51–100 µm). The microvascular flow index (MFI) was calculated as the sum of each quadrant score divided by the number of quadrants in which the vessel type was detected. The final MFI was an average based on results from a minimum of 12 quadrants (i.e., three regions, four quadrants per region) that were derived from the overall blood flow determined as described above for blood vessels assigned to each diameter-cohort [16,17]. The total vessel density (TVD) was calculated using the AVA software package for small vessels (primarily capillaries) with a cut-off diameter of <20 μm. The proportion of perfused vessels (PPV) among small vessels was determined by dividing the length of the perfused small vessels by the total length of all small vessels. The perfused vessel density (PVD) of the small vessels was calculated by measuring the density of all perfused small vessels within the field of view (computed as the proportion of perfused vessels multiplied by the total vessel density). The De Backer score was calculated as the number of small vessels crossing three equally spaced vertical and horizontal lines in the image field divided by the total length of the lines [15,18].

### 2.2. Evaluation of the Glycocalyx

The human glycocalyx is extremely fragile, thus visualizing this structure in vivo is highly challenging. However, the region that is partially accessible to flowing red blood cells at the luminal side has been characterized and defined as the perfused boundary region (PBR) [19]. Degradation of the glycocalyx results in the deeper penetration of the RBCs towards the endothelium and thus in an increased PBR [20].

An SDF video microscope (Microscan, MicroVision Medical BV, Amsterdam, The Netherlands) attached to a GlycoCheck analyzer (GlycoCheck BV, Maastricht, The Netherlands) was used to evaluate PBRs in the sublingual microvasculature. Ten image sequences, each including 40 frames, were recorded in different areas at this site and the PBR was calculated automatically. The RBC column was automatically measured in 3000 vascular segments. For each segment, 840 radial intensity profiles were captured to measure the RBC column width. The PBR was estimated as the distance from the median (P50) RBC column width to the (estimated) outer edge of the RBC-perfused lumen. As described in previous publications [21,22], the vessel segments were classified in 1 μm-wide diameter classes. Median PBR values were determined for each diameter class before calculating the average PBR over a set of diameters ranging from 5 to 25 μm. As described above, vessel diameters were categorized into groups that included (small) 5–9 μm, (medium) 10–19 μm, and large (20–25 μm) to facilitate further analysis.

### 2.3. Statistical Analysis

Data were analyzed with Statistical Package for Social Sciences (SPSS 26 for Windows, Chicago, IL, USA). Statistical significance was determined using non-parametric tests in consideration of the small sample size. The Mann–Whitney U test was used to compare variables between patients and controls. Correlations between clinical and microvascular parameters were assessed using a Spearman rank correlation coefficient. The tests used were two-sided, and a *p*-value of <0.05 was considered significant. Our study was powered to detect moderate correlations (Spearman correlation coefficient = 0.5) between PBR and microcirculatory parameters in the septic cohort with 80% power associated with a two-sided alpha of 0.05. Data are presented as median and 25–75th percentiles.

## 3. Results

Clinical, demographic, and microcirculatory characteristics of the septic patients enrolled in our study are shown in Table 1. The predominant source of infection was the abdomen, which accounted for 42.9% of the patient cohort. Others included pneumonia (28.6%), urinary tract infection (10.7%), and others (17.9%). The median SOFA score was 9, indicative of severe disease. All patients were mechanically ventilated and receiving vasopressor therapy at the time of inclusion.

**Figure 1:**
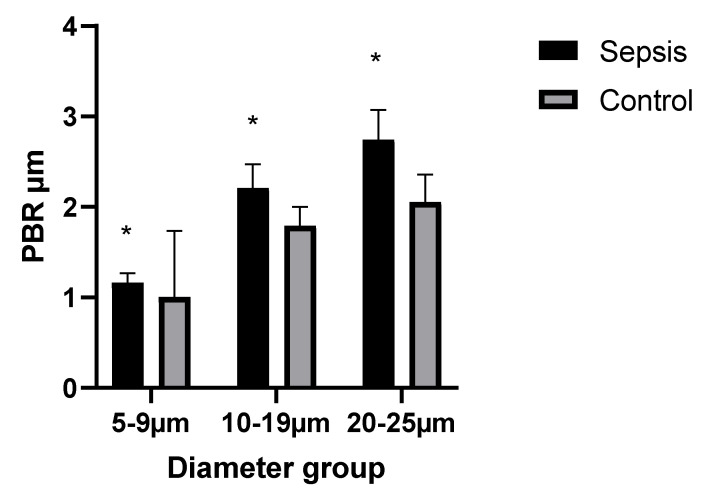
Comparisons between the perfused boundary regions (PBRs) of microvessels in the three diameter groups in septic patients and healthy volunteers. Data are presented as mean ± standard deviation; * *p* < 0.05 compared to healthy subjects.

An assessment of the sublingual glycocalyx is presented in Table 2. Compared to healthy subjects, septic patients demonstrated significantly increased PBRs in all blood vessel diameter groups (*p* < 0.001), indicating reduced glycocalyx thickness (Figure 1).

**Figure 2:**
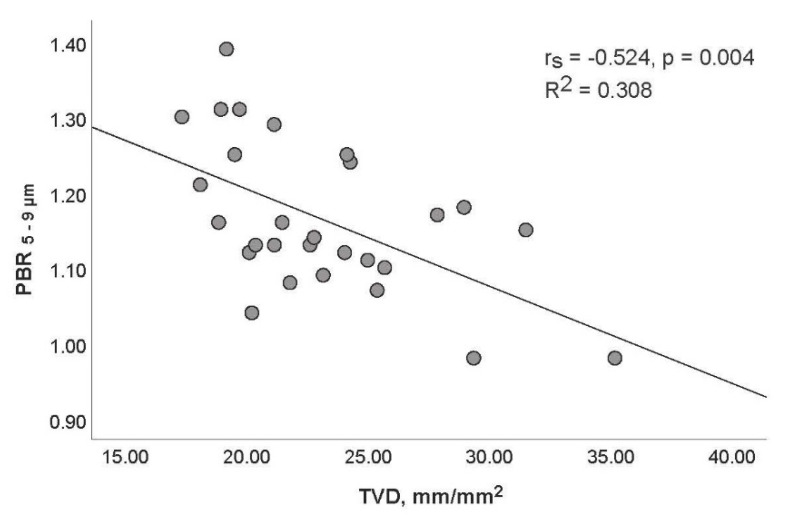
Relationship between perfused boundary region (PBR) detected in microvessel diameter group 5–9 μm (PBR_5–9_) and total vessel density (TVD) of small vessels in septic patients.

**Figure 3:**
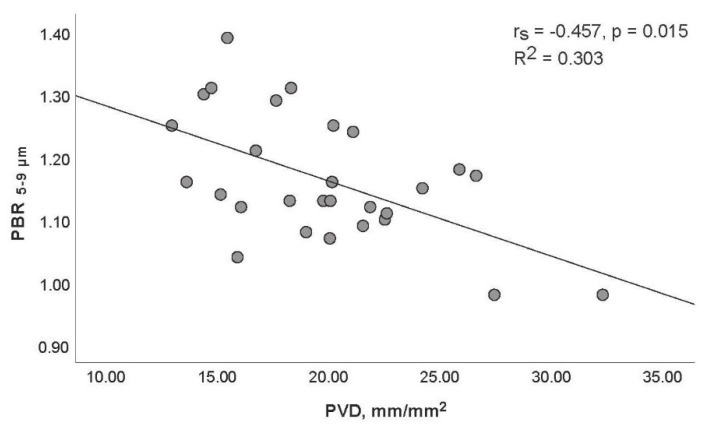
Relationship between perfused boundary region (PBR) detected in microvessel diameter group of 5–9 μm (PBR_5–9_) and perfused vessel density (PVD) of small vessels in septic patients.

The lengths (in µm) of the PBRs detected in the smallest capillaries (diameter class 5–9 µm) correlated moderately (and inversely) with TVD (rs = −0.524, *p* = 0.004; Figure 2) and PVD (rs = −0.457, *p* = 0.015; Figure 3).

PBRs detected in the larger microvessels (20–25 µm) correlated directly with serum lactate levels (rs = 0.45, *p* = 0.01) and correlated inversely with base excess (BE) (rs = −0.41, *p* = 0.02) and bicarbonate levels (rs = −0.40, *p* = 0.027).

Correlations between PBRs detected in the various diameter groups were also observed. Specifically, PBR5–9 correlated directly with PBR10–19 (rs = 0.41, *p* = 0.03) and PBR10–19 correlated directly with PBR20–25 (rs = 0.724, *p* < 0.001). However, we identified no correlation between PBR5–9 and PBR20–25.

We also found no correlations between PBR and MAP, heart rate (HR), C-reactive protein (CRP), interleukin (IL-6), APACHE II scores, or SOFA scores.

## 4. Discussion

Collectively, our results document correlations between decreased glycocalyx thickness and microcirculatory impairments observed in septic patients. Our results suggest a moderate correlation between PBR in small capillaries (PBR_5–9_) and TVD, as well as between PBR_5–9_ and PVD in patients diagnosed with sepsis. This finding is consistent with the results of an experimental animal study performed by Cabrales et al. [23]. In this study, glycocalyx damage induced by hyaluronidase from *Streptomyces* was accompanied by a significant redistribution of RBC flow in a capillary network; this led to a significant increase in RBC empty flowing capillaries. These findings were also accompanied by a corresponding decrease in functional capillary density and increased capillary hematocrit. Taken together, the results suggested that damage to the glycocalyx results in suboptimal capillary perfusion. Interestingly, alterations in glycocalyx integrity had no significant impact on blood flow in larger vessels at the baseline or in response to the maximal effects of hyaluronidase treatment [23].

Marechal et al. [24] found that endotoxin-induced microvascular dysfunction was associated with increased oxidative stress and loss of EG in an experimental study carried out in rodents. These results were confirmed in a study performed with human volunteers by Nieuwdorp et al. [25] Reductions in glycocalyx thickness following endotoxin infusion were observed in this latter study in the capillaries of 5–7 μm diameter concomitantly with microcirculatory alterations. Relationships between reduced EG thickness and alterations in the microvasculature were also demonstrated in cardiopulmonary bypass patients [26,27,28] and individuals exhibiting chronic oxidative stress [19].

By contrast, several studies reported a dissociation between PBR and microcirculatory flow parameters during sepsis. In an interventional study that examined the acute impact of leukocyte-depleted RBC transfusions in septic patients, Donati et al. [29] found that the median PBR values remained unchanged after this intervention. Nonetheless, improved microvascular parameters were reported in the treatment groups. However, it is critical to recognize that the time needed to restore standard dynamically relevant EG thickness in vivo can be up to seven days; by contrast, microcirculatory parameters can change extremely rapidly [30,31,32]. These results highlight the multifactorial basis of microvascular alterations under conditions of glycocalyx shedding. Similarly, Rovas et al. [6] also reported that there were no correlations between damage to the glycocalyx and microcirculatory parameters based on results from a comprehensive observational study that enrolled patients with resuscitated sepsis. However, the relatively long duration of sepsis at the time of recruitment to this study should be noted. Timing may be an essential factor underlying this discrepancy, as microvascular alterations tend to be more severe at the earlier stages of the disease and more moderate in persistent sepsis [10]. Additionally, differences in the severity of illness and the sources of infection could contribute to these results. In our study, 50% of the patients were diagnosed with septic shock, compared to only 10% in the Rovas et al. study. Similarly, pneumonia was the leading cause of sepsis in the Rovas et al. study, while abdominal infection was more prevalent in our patient cohort. One plausible explanation is that the threshold for and timing of glycocalyx damage differs in various tissues. This could result in the spatiotemporal uncoupling of damage to the glycocalyx and alterations in microcirculatory parameters [6,25,33]. Moreover, in a subsequent study, Rovas et al. [7] reported that correlations between glycocalyx damage and microcirculation parameters might improve when the flow-dependency of the glycocalyx and capillary density are taken into account.

Our findings revealed a correlation between serum lactate levels and other indices of metabolic acidosis with PBR in microvessels with a diameter of 20–25 µm. This finding might be explained by the association of smooth muscle tone of the precapillary vessels and autoregulation of blood flow. Local metabolic effects are mainly detected in terminal arterioles; the EG plays the leading role in this mechanotransduction response in septic patients [34]. Loss of the glycocalyx in response to pathological conditions, for example, inflammation, can lead to impaired local regulation of blood flow, loss of flow-dependent vasodilation, and tissue hypoxia [35].

We also identified an intergroup correlation between PBR in microvessel diameter groups that include 5–9 µm and 10–19 µm, but not between the smaller capillaries and the 20–25 µm diameter group. These findings suggest that numerous mechanisms are involved in promoting glycocalyx alterations in capillaries of different diameter classes. These findings also suggest that critical autoregulatory processes take place at the intersection of capillaries at 10–19 µm and 20–25 µm in diameter.

We identified no correlations between microcirculatory changes, glycocalyx thickness, and systemic hemodynamic parameters (for example, MAP). The results do not contradict previous findings that described the loss of hemodynamic coherence in septic patients [36]. Similarly, we observed no correlation between these parameters and either SOFA score or APACHE II score. Interestingly, Rovas et al. [7] reported that the combination of flow-dependent capillary density and flow-dependent glycocalyx parameters can be used to generate an overall Microvascular Health Score (MVHS) that correlates effectively with the SOFA score.

Our findings that detail the extent of glycocalyx damage in different capillary diameter groups are consistent with previous studies. Specifically, our results confirmed findings indicating that the glycocalyx becomes thinner in vessels of all diameters in response to septic conditions [6,7].

We acknowledge some limitations of our study. First, the study was carried out at a single center and involved a small sample size. However, we do note that the patients enrolled in our study were subject to strict inclusion criteria and a limited 24 h timeframe. Second, we have used manufacturer-defined vascular diameters as our classification scheme. We recognize that this may not necessarily reflect physiological processes. Third, the use of both second and third-generation video microscopy (SDF, IDF) might contribute to sampling error and have an impact on the results. However, real-time assessment of the MFI showed a good agreement between subsequent examinations, analogous to findings presented in other studies [6]. Fourth, IDF yields higher quality images and thus can detect more vessels than are typically identified with SDF. However, given that the GlycoCheck system can reliably recognize and analyze RBC flux and thus can be used to calculate the PBR even in the capillaries as small as 4 μm in diameter, this difference is most likely negligible.

And finally, the dynamic and adaptive nature of changes in the glycocalyx cannot be excluded from our consideration. However, from a functional point of view, all possible modifications of the nanomechanical properties of the endothelial surface are important and EG that has collapsed or been shed may be a strong promoter of adverse effects with a profound impact on the microvascular system [37].

## 5. Conclusions

Our findings confirmed damage of the glycocalyx in the microvasculature of all diameter ranges in patients with sepsis compared to healthy control subjects. Our findings demonstrated a direct relationship between damage to the EG in the smallest capillaries and sepsis-associated impairments in the microcirculation. The mechanisms underlying these findings and the precise timing of this relationship remain to be determined.

## Figures and Tables

**Table 1:** Baseline characteristics of septic patients. Data are presented as median, 25th, and 75th percentiles as relevant.

Variable	Septic Patients
Number of participants	28
Female sex; n (%)	9 (32)
Age (years)	65 (58–80)
Body mass index (BMI; kg/m^2^)	25.0 (21.0–27.8)
Time to inclusion (hours)	14 (6–24)
SOFA score	9 (8–11)
Mechanical ventilation (n (%))	28 (100)
Vasopressors (n (%))	28 (100)
Norepinephrine	
Dose (μg/kg/min)	0.23 (0.12–0.37)
Septic shock (n (%))	14 (50)
In-hospital mortality (n (%))	12 (42.9)
APACHE II score	17.50 (14.25–22.00)
Mean arterial pressure (MAP; mmHg)	75 (63–83)
Heart rate (beats/min)	106 (95–115)
Cardiac index (CI; L/min/m^2^)	3.2 (2.0–4.4)
**Laboratory data**	
C-reactive protein (CRP; mg/L)	252 (133–415)
Interleukin (IL)-6 (pg/mL)	875 (450–2087)
pH	7.301 (7.261–7.398)
Partial pressure of oxygen (pO_2_, mmHg)	110 (80–144)
Serum lactate (mmol/L)	2.3 (1.4–3.8)
Central venous oxygen saturation (ScvO_2_,%)	76.5 (70.8–78.8)
**Microcirculation data**	
Total vessel density (TVD, mm/mm^2^)	22.2 (19.8–25.3)
Perfused vessel density (PVD, mm/mm^2^)	19.9 (15.9–22.3)
Proportion of perfused vessels (PPV, %)	87.8 (79,0–91.7)
Microvascular flow index (MFI)	1.96 (1.52–2.17)
De Backer score	14.2 (12.2–16.5)

**Table 2:** Perfused boundary region (PBR) of healthy subjects and septic patients. Data are presented as median, 25th and 75th percentiles.

Endothelial Glycocalyx (EG)	Healthy Subjects	Septic Patients	*p*-Value
PBR; 5–25 (μm)	1.70 (1.53–1.84)	2.10 (2.00–2.30)	<0.001
PBR; 5–9 (μm)	1.00 (0.97–1.06)	1.14 (1.10–1.25)	<0.001
PBR; 10–19 (μm)	1.79 (1.70–2.00)	2.24 (2.06–2.40)	<0.001
PBR; 20–25 (μm)	2.06 (1.76–2.29)	2.75 (2.52–2.94)	<0.001
